# Catechin tuned magnetism of Gd-doped orthovanadate through morphology as T_1_-T_2_ MRI contrast agents

**DOI:** 10.1038/srep34976

**Published:** 2016-10-18

**Authors:** Tamilmani Vairapperumal, Ariya Saraswathy, Jayasree S. Ramapurath, Sreeram Kalarical Janardhanan, Nair Balachandran Unni

**Affiliations:** 1 Chemical laboratory, CSIR-Central Leather Research Institute, Adyar, Chennai, 600 020, India; 2 Department of Physics, NSS College, Pandalam 689501, Kerala, India; 3 Biophotonics and Imaging Lab, Sree Chitra Tirunal Institute for Medical Sciences and Technology, BMT Wing, Poojappura, Trivandrum 695012, India

## Abstract

Tetragonal *(t)-*LaVO_4_ has turned out to be a potential host for luminescent materials. Synthesis of *t-*LaVO_4_ till date has been based on chelating effect of EDTA making it not ideal for bioimaging applications. An alternative was proposed by us through the use of catechin. In recent times there is interest for new MRI contrast agents that can through appropriate doping function both as MRI contrast and optical/upconversion materials. It is generally believed that under appropriate doping, *t-*LaVO_4_ would be a better upconversion material than monoclinic (*m)-*LaVO_4_. Based on these postulations, this work explores the use of gadolinium doped *t-*LaVO_4_ as an MRI contrast agent. From literature, gadolinium oxide is a good T_1_ contrast agent. Through this work, using catechin as a template for the synthesis of Gd doped *t-*LaVO_4_, we demonstrate the possible use as a T_1_ contrast agent. Interestingly, as the catechin concentration changes, morphology changes from nanorods to square nanoplates and spheres. In this process, a switch from T_1_ to T_2_ contrast agent was also observed. Under optimal concentration of catechin, with a rod shaped Gd doped *t-*LaVO_4_ an r_2_/r_1_ value of 21.30 was observed. Similarly, with a spherical shape had an r_2_/r_1_ value of 1.48 was observed.

In recent years, efforts to couple imaging modalities such as optical and magnetic resonance imaging have met with success. Such coupling brings to the fore the advantages of both the methods, say, with respect to imaging resolution and penetration depth^1^,^2^. Magnetic resonance imaging (MRI) is a non-invasive method to diagnose diseases owing to its high spatial resolution and good soft-tissue contrast. It works either by shortening the longitudinal (T_1_) or the transverse (T_2_) relaxation time of water protons. MRI contrast agents (CAs) can be classified as either positive (T_1_) CAs or negative (T_2_) CAs. Typically, MRI signal obtained by T_2_ CAs is easily confused with other artifact signals, like calcification, bleeding, and metal deposits, Etc. Generally positive T_1_ CAs is widely used as extracellular, hepatobiliary, and blood pool agents in medical imaging. Positive T_1_ CAs gained its advantage for a bright MR image, high longitudinal relaxation rate, low cytotoxicity, and low intake dose. Currently, Gadolinium chelates like Gd-DOTA and Gd-DTPA are used as T_1_ CAs but release a certain amount of free Gd ions, which inhibit calcium channels which leads to cardiovascular and neurologic toxicity. Also, Gd-based inorganic nanoparticles, such as carbonate (Gd_2_ (CO_3_)_3_), fluoride (GdF_3_, NaGdF_4_), oxide (Gd_2_O_3_), and vanadate (GdEuVO_4_) were investigated^3^. The advantage of doping Gd ions into the host crystal structure lies in a very low leaching of free Gd ions and even more stable than Gd based MRI contrast agents^4^. In Gd doped host lattices, surface Gd^3+^ ions offer all seven of its unpaired electron for water hydration by Inner sphere contribution, which cooperatively induce the longitudinal relaxation of water proton. Gd^3+^ chelates can only offer one hydrate position since their other six unpaired electrons are coordinated by chelates. This synergistic effect enhances relaxivity value of Gd doped host lattices than Gd chelates^3^. Nanostructures based on Gd^3+^ doped NaYF_4,_ codoped with Yb^3+^/Er^3+^ has been reported for upconversion imaging coupled with MRI^5^. Unfortunately, fluorides tend to be hygroscopic and have less favorable chemical and photophysical stabilities^6^.

Lanthanide orthovanadates are potential hosts for luminescent materials, when in appropriate matrices. LaVO_4_ exists in two phases, viz., the monoclinic, monazite structure and tetragonal zircon structure. La^3+^ generally prefers the monazite structure as the thermodynamically stable state. While *m-*LaVO_4_ is not a suitable host for luminescent activators, *t*-LaVO_4_ is a promising phosphor. This variability in the properties of the polymorphs, had created an extensive interest in selective synthesis and phase change processes^7^. Through the years, hydrothermal method based on EDTA has emerged as an effective way to synthesize the metastable *t-*LaVO_4_^8^.

Though *t-*LaVO_4_ has emerged as a potential luminescent material, the success of the same as an upconversion phosphor has been limited. It is only recently that Singh *et al*.^9^ and Zheng *et al*.^10^ reported the upconversion properties of Yb^3+^/Er^3+^ doped *t*-LaVO_4_. No such reports were found in the literature for Gd-doped *t*-LaVO_4_. One of the challenges for the bioimaging applications of the orthovanadate is in the use of EDTA for the selective synthesis of *t-*LaVO_4_. EDTA is known to bring about cytotoxicity^11^.

Encouraged by the need to overcome the drawbacks, we for the first time report catechin assisted *t-* Gd-doped lanthanum vanadate (GL) nanoparticles with varying morphologies as T_1_ contrast agents. Catechin possesses a large number of phenolic hydroxyl groups susceptible for metal chelation, and its biocompatibility led to the choice compared to that of other existing additive systems like EDTA, and citric acid. As structure of catechin is pH dependent, it is stable in highly acidic solution and unstable in neutral or alkaline solution. In general, Catechin is absorbed from the human intestinal tract, largely metabolized and distributed as conjugated derivatives in blood, and that these forms are excreted in urine^12^. Catechin is known for its superior hydrophilic antioxidant property because of its higher number of hydroxyl groups and retards lipid oxidation. It can scavenge hydroxyl, peroxyl, and 2,2-diphenyl-1-picrylhydrazyl (DPPH) radicals. The thermal stability of catechin in the presence of oxygen is 227 °C and weight loss from 50 to 110 °C is due to water evaporation^13^. Catechin interacts with plasma proteins through different covalent and noncovalent bonds (i.e., hydrogen bonding, π-bonding, hydrophobic, and ionic interactions), and brings about significant changes in structure, physicochemical properties, and the activity of proteins^14^. It has a strong affinity with lipid bilayers, which facilitates their entry into cancer cells^15^. Since catechin is known for its beneficial effects like antioxidative, anticancer, anti-inflammatory and antithrombogenic activities^7^,^11^,^16^ it is expected that catechin modulated hydrothermal synthesis could offer a 3-fold advantage, viz., phase, morphology, and magnetization directed synthesis.

## Results and Discussion

**Figure 1:**
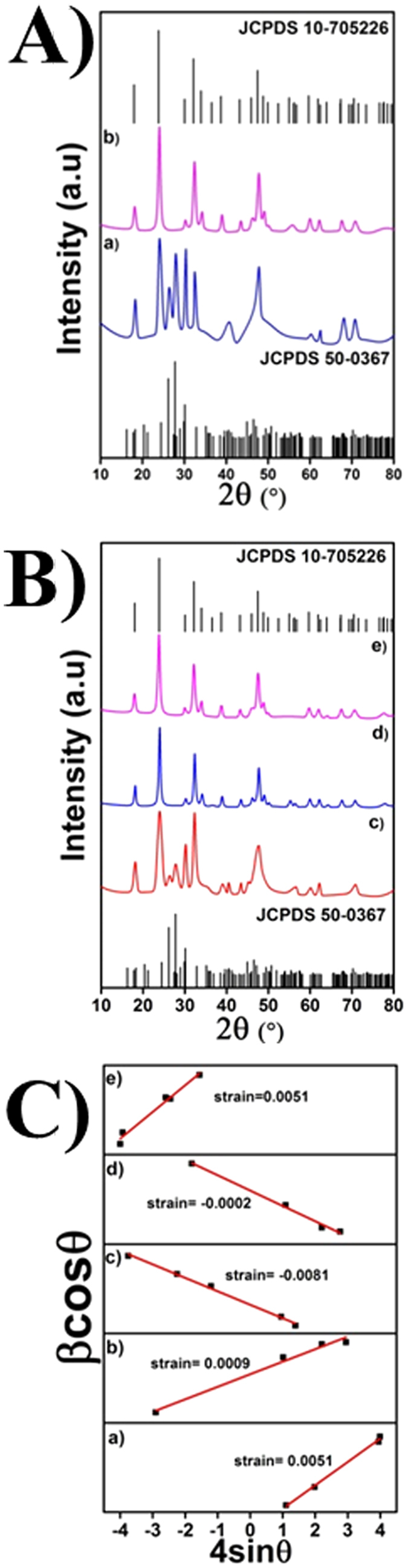
XRD pattern of GL nanoparticles (**A**) without ***cat*** (**a**) and ***cat*** (**b**, [cat^4+^]/[La^3+^] = 1:0.05) (Experimental conditions: T = 210 °C, t = 4 h, pH = 7), (**B**) [cat^4+^]/[La^3+^] = 1:0.01(c), [cat^4+^]/[La^3+^] = 1:0.05(d) and [cat^4+^]/[La^3+^] = 1:1 (e) (Experimental conditions: T = 180 °C, t = 24 h) and (**C**) Corresponding W-H plot.

**Figure 2:**
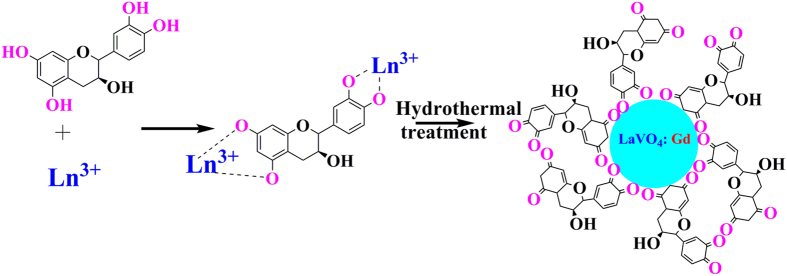
Depicts the formation of GL nanoparticles using catechin hydrate.

**Figure 3:**
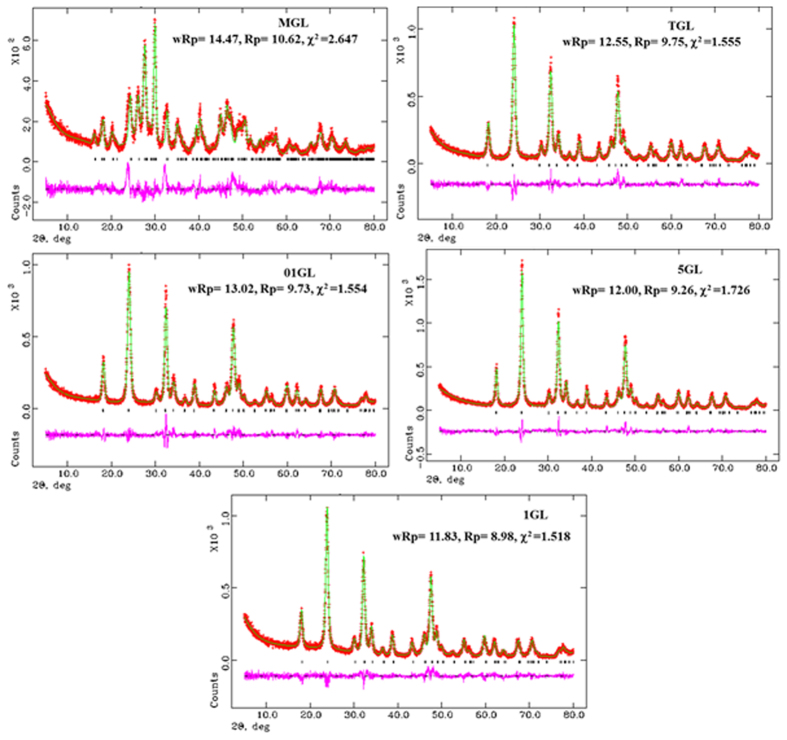
Final Rietveld XRD data plot of GL nanoparticles with the values of agreement factors and χ^2^ (red, observed; green, calculated; black, vertical bars – positions of the Bragg reflections; pink, difference between observed and calculated intensities).

The role of catechin hydrate (***cat***) in polymorph selection for a doped system can be seen from Fig. 1. The product obtained from the hydrothermal treatment of La(NO_3_)_3_.6H_2_O, Gd(NO_3_)_3_.6H_2_O and Na_3_VO_4_ in the absence of catechin, was well indexed to *m*-LaVO_4_ (JCPDS No. 500367) with a space group (p21/n)], cell parameters a = 7.043 Å; b = 7.279 Å; c = 6.721 Å and cell volume = 333.071 Å^3^ (Fig. 1A(a)). Incorporation of ***cat*** as ligand accelerates the formation of tetragonal phase with cell parameters a = b = 7.4578 Å; c = 6.5417 Å and cell volume = 363.841 Å^3^ [JCPDS no. 10–705226; space group I41/amd (141)] as shown in Fig. 1A(b). It can thus be seen that at an appropriate concentration of ***cat,*** the formation of pure *t-*LaVO_4_ without the presence of impurity phases such as *m*-LaVO_4_ is possible, similar to our earlier observations (Fig. 1B). Catechin act as the capping as well as stabilizing agent by the interaction of Ln^3+^ (Ln = La, Gd) with phenolic OH groups at 5, 7, 3′, 4′ positions (Fig. 2)^17^. Well-resolved peaks, as can be seen in Fig. 1B, indicates a highly crystalline nature, alongside lower defects, the added advantage being the use of low hydrothermal treatment temperature (180 °C). Sharp peaks with even peak profiles coupled with highly crystalline nature is an indication of smaller crystallite sizes. This can be further confirmed from the crystallite size calculated by Debye-Scherer formula. Cell parameters thus obtained are provided in Table 1. Lattice strain calculated by Williamson-Hall (W-H) method employing the plot in Fig. 1C, further demonstrates the formation of the metastable state. Crystal structure of five GL nanoparticles was established from Rietveld structural refinement of slow scan powder XRD data (Fig. 3).

**Figure 4:**
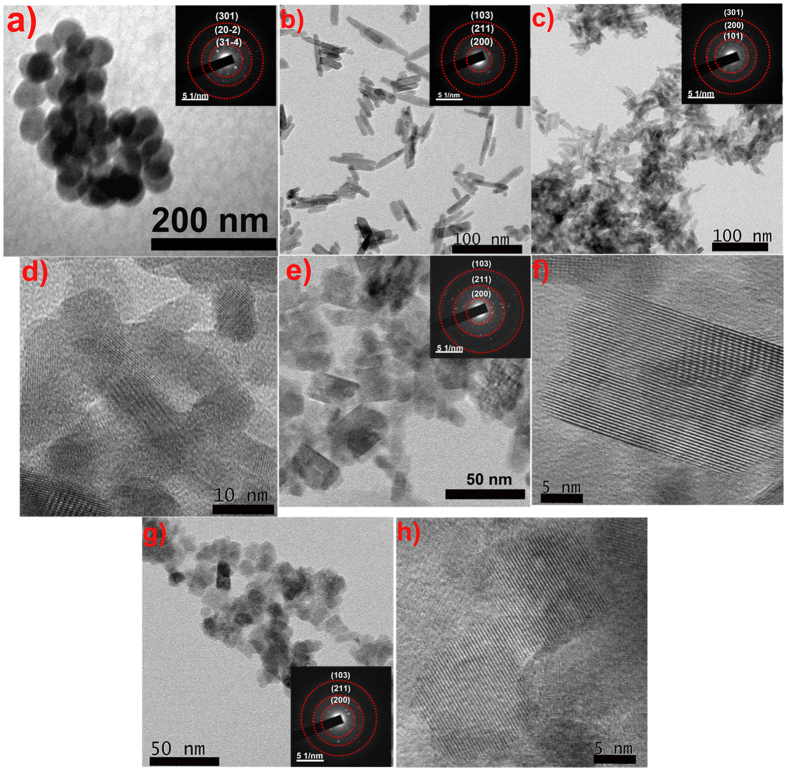
TEM Image for MGL (**a**), TGL (**b**), 01GL (**c**), 5GL (**e**) and 1GL (**g**) and HRTEM images of 01GL (**d**), 5GL (**f**) and 1GL (**h**) nanoparticles [Inset represents the corresponding SAED pattern].

An indication of the morphological features of the nanoparticles was obtained from transmission electron microscopic images (TEM). Gd doped *m-*LaVO_4_ (marked as ***MGL*** for better representation) nanoparticles were spherical with an average size of around 40 nm (Fig. 4a). In the presence of ***cat***, *t*-LaVO_4_, with a rod-like morphology (mean length of 30 nm, mean diameter of 9 nm, the aspect ratio of 3) was obtained (Fig. 4b, marked as ***TGL*** for better representation). Morphological changes with varying ***cat*** concentration are depicted in Fig. 4c–h, where at 0.01 mM ***cat*** (marked as ***01GL**),* nanorods with the length of 20–30 nm and diameter of 7 nm, the aspect ratio of 3 was observed. That the LaVO_4_ existed in the tetragonal form was confirmed by comparing the lattice fringes (d = 2.2388 Å), with the (301) plane of standard *t-*LaVO_4_. At 0.05 mM ***cat,*** the nanoparticles (marked as ***5GL***) existed as irregular rectangular NPs with the length of 14–25 nm and diameter of 12–22 nm respectively. Corresponding high-resolution images and SAED pattern (inset of Fig. 4e) showed the single crystalline character that could easily be correlated to standard *t*-LaVO_4_. As the concentration of ***cat*** is increased (0.1 mM, marked as ***1GL***), a thermodynamically stable spherical morphology is obtained, with a diameter in the range of 12 nm. HRTEM image of ***1GL***, indicated well-defined 2D lattice planes with d spacing of 1.9231 Å indexed to (312) plane [JCPDS-10705226]. SAED (Fig. 4g inset) of ***1GL*** shows a single crystalline diffraction pattern, indexed to (200), (211) and (103) planes of *t-*LaVO_4_.

**Figure 5:**
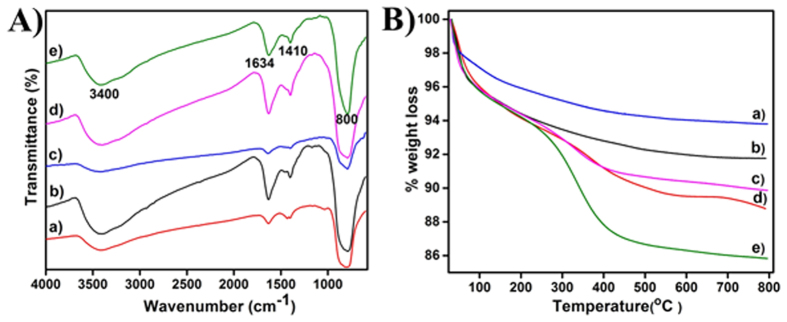
FTIR spectrum (**A**) and TGA (**B**) for (a) MGL, (b) TGL, (c) 01GL, (d) 5GL and (e) 1GL nanoparticles.

Gd doped LaVO_4_ nanoparticles obtained by employing ***cat*** as a chelating agent was analysed by TGA and FTIR. The spectra presented in Fig. 5 provide an indication of 8–14% organic matter being still present in the residue. TGA profile of nanoparticles showed dehydration of water around 100 °C^18^, followed by further weight loss between 200–450 °C, corresponding to catechin, as reported elsewhere^11^. From the FTIR spectrum, it can be seen that the hydroxyl groups in phenolic and water molecules appear as broad absorption band around 3400 cm^−1^, the doublet bands observed at 1634 and 1410 cm^−1^ are related to the localized vibration of VO_4_ groups and C = C stretching frequencies from the aromatic rings. A band around 800 cm^−1^, corresponds to the characteristic peak of V-O from VO_4_ groups^19^ and C-O-C group in catechin molecules^7^,^16^.

**Figure 6:**
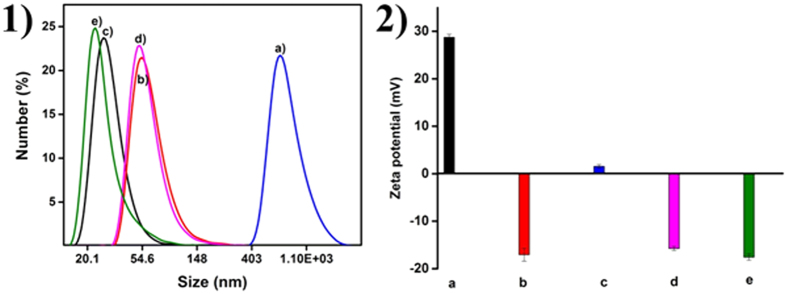
DLS (**1**) and Zeta potential (**2**) for (a) MGL, (b) TGL, (c) 01GL, (d) 5GL, and (e) 1GL nanoparticles.

Dynamic Light Scattering (DLS) in back scattering geometry was performed to determine the hydrodynamic size of the Gadolinium doped LaVO_4_ nanoparticles. Figure 6a presents the hydrodynamic diameters of the nanoparticles dispersed in double distilled water (1 mg/5 mL). Assuming a spherical geometry, DLS measurement has been carried out. The nanoparticles demonstrated polydisperse behavior (PDI = 0.590) with a number average diameter of 798 nm, attributable to the nonuniform size and nanoparticle aggregation. Number average diameter of nanoparticles in the presence of catechin was low, suggesting that strong capping effect had rendered uniform size. Polydispersity index (PDI) of 0.24, 0.33, 0.27, and 0.27 respectively indicate a near monodisperse distribution.

**Figure 7:**
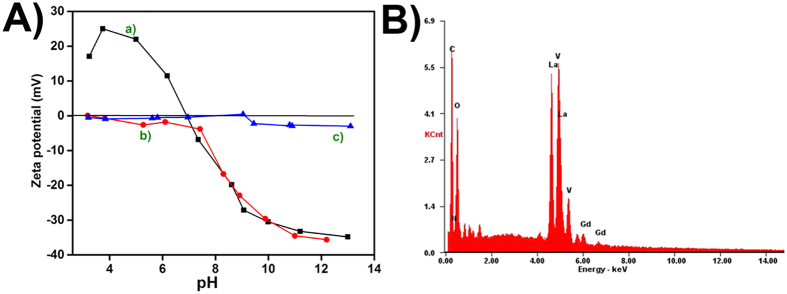
Zeta potential at various pH values for (**A**) 01GL (a), 5GL (b) and 1GL (c) and EDAX spectrum (**B**) nanoparticles.

Zeta potential values provide information on the stability of the nanoparticles in a given environment^20^,^21^,^22^. The pH at which charge of the nanoparticle and its immediate surroundings (double layer) becomes zero (point of zero charge (PZC)) was monitored (Fig. 7A) and it was found that PZC changes from 6.91 to 3.25 and then increases to 7.93. This shift in PZC to a higher pH value could be attributed to the presence of catechin molecules on the nanoparticle surface. In the absence of ***cat*** the Gd doped LaVO_4_ synthesized had a positive charge (28.7 ± 0.5 mV) at pH 8, which shifted to a negative value of −17.1 mV on treatment with ***cat***, indicating that the surface of the nanoparticles was covered with organic moiety^7^. At ***cat*** concentration of 0.05 to 0.1 mM, the zeta potential values of the doped vanadates were more or less constant at around −16 ± 1 mV. A high negative zeta potential, as observed in this study is an indication of the stability of the Gd doped *t*-LaVO_4_ making it viable for potential biological applications. A zeta potential value of 1.56 mV observed for the nanoparticles at a ***cat*** concentration of 0.01 mM could be attributed to the fact that cationic Ln^3+^ ions were coordinated to phenolic –OH groups in catechin, resulting in the neutralization of the negative surface charge. The presence of Gd, V, La, O and C was confirmed from the EDAX spectra (Fig. 7B). The atomic ratio for La^3+^ and Gd^3+^ was determined as 0.92 and 0.06 respectively, approaching the theoretical value. Gadolinium concentration in MGL, TGL, 01GL, 5GL, 1GL was found to be 2.83, 0.574, 7.712, 10.32, 1.89 mg/kg from ICP-OES measurements.

**Figure 8:**
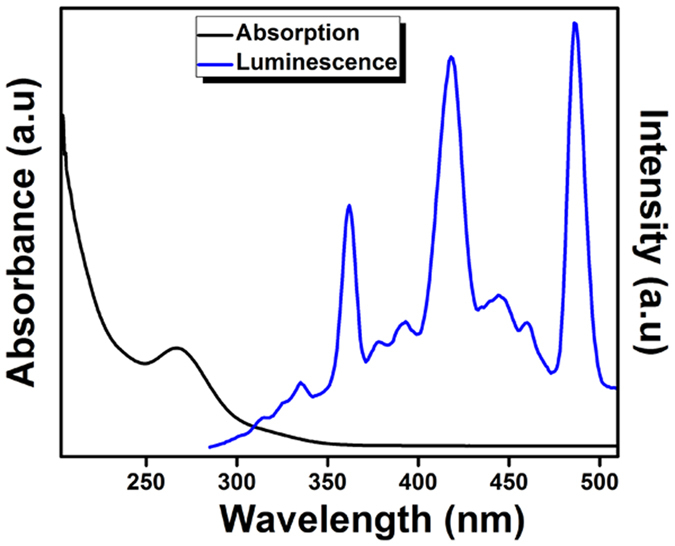
Absorption (Black line) and luminescence spectra (Blue line) for Gd doped *t-*LaVO_4_ nanoparticles.

Luminescence properties of Gd doped *t-*LaVO_4_ nanoparticles is shown in Fig. 8. The strong absorption band around 267 nm corresponds to charge transfer from the oxygen ligands to central vanadium metal in VO_4_^3−^ groups^23^. Gd doped *t-*LaVO_4_ exhibit emission peak at 334 nm corresponds to ^6^P-^8^S transition of Gd^3+^ 24 whereas emission group lines between 360 and 520 nm, corresponds to VO_4_^3−^ transitions^25^. MTT assay^26^ was performed in order to understand the effect of Gd- doped tetragonal LaVO_4_ on cell viability and toxicity. The results of MTT assays are given in Supporting Figure S1. The results showed that treatment of HaCaT cells with Gd- doped tetragonal LaVO_4_ did not affect the viability of the cells. The cells did not show significant toxicity at Gd- doped tetragonal LaVO_4_ concentration as high as 100 μg. The results are consistent with the microphotographs which revealed that the cell structure and morphology were not affected at a concentration as high as 100 μg (Figure S1).

**Figure 9:**
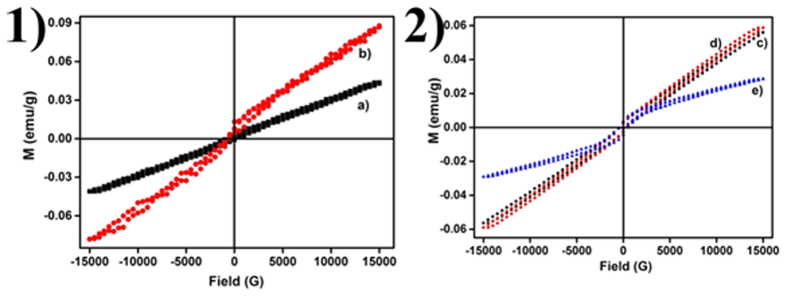
Magnetization curves of GL nanoparticles at 300 K **1**) without ***cat* (a)** and ***cat* (b**, [cat4−]/[La3 +] = 1:0.05) (Experimental conditions: T = 210 °C, t = 4 h, pH = 7), 2) [cat4−]/[La3+] = 1:0.01 (**c**), [cat4−]/[La3+] = 1:0.05 (**d**) and [cat4−]/[La3+] = 1:1 (**e**) (Experimental conditions: T = 180 °C, t = 24 h).

Magnetization curves measured in the applied magnetic field sweeping from −15 to 15 kG at 300 K, in the presence and absence of ***cat*** is presented in Fig. 9. The samples were found to be paramagnetic (**P**) with a high paramagnetic moment, attributable to a higher number of unpaired electrons in the half-filled 4f^7^ outermost orbital of the Gd^3+^ ion^27^ with M_s_ value of 42.4, 18.32, 56.19 and 58.87 emu/g respectively. It is known that saturation magnetization of nanoparticles can be affected by structural defects; crystallite size, shape and amount of catechin present on the LaVO_4_ nanoparticle, i.e. surface state^28^,^29^,^30^,^31^,^32^,^33^. At higher ***cat***, nanoparticles become superparamagnetic (**SP**), with a low M_s_ value of 0.0288 emu/g^34^,^35^. This shift in M_s_ may be due to strong co-ordination ability of the catechin molecules^36^. Comparatively, saturation magnetization on a per-gram basis is lower may be due to the lack of full spin alignment in the particles i.e. spin canting effect induced by the high mass of the nonmagnetic catechin coating on the nanoparticle surface^37^,^38^. This clearly establishes that catechin molecules play a key role on magnetic properties of nanoparticles. Table 2 records the coercivity (H_c_), saturation magnetization (M_s_), remnant magnetization (M_r_) and squareness ratio values for the nanoparticles. The ratio of M_r_ to M_s_ is almost found to be constant for all paramagnetic material.

**Figure 10:**
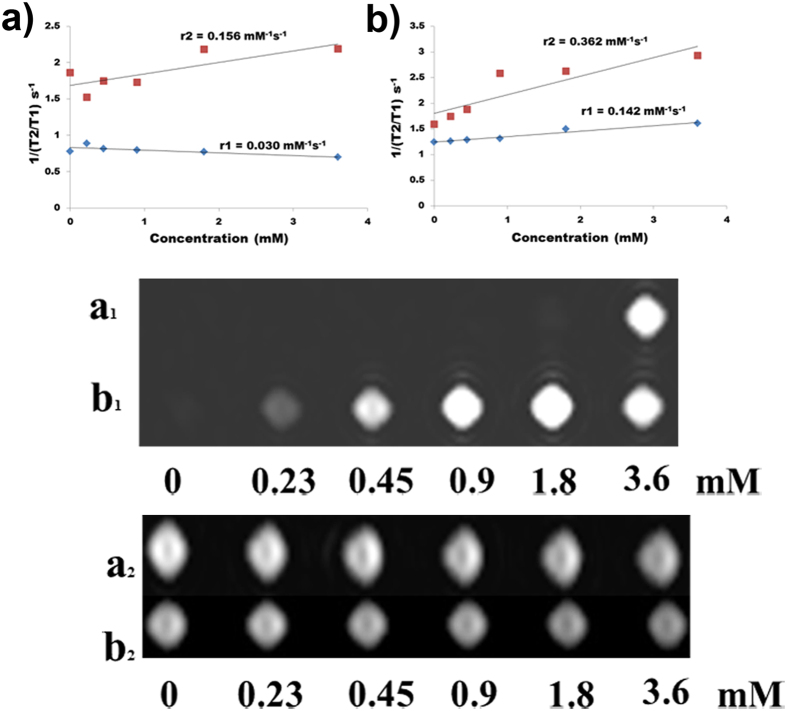
Linear fit plot employed for the calculation of r_1_ and r_2_: T_1_- and T_2_-weighted phantom images of GL nanoparticles with different concentrations where (**a**) MGL and (**b)** TGL. Subscript 1 and 2 represents T_1_ and T_2_ relaxivity.

We examined the possibility of developing P-Gd and SP-Gd as MRI bimodal contrast agents. To evaluate the MRI imaging properties, series of gadolinium doped LaVO_4_ nanoparticles in aqueous solutions containing different concentrations (3.6, 1.8, 0.9, 0.45, 0.23 and 0 mM) were prepared for MRI phantom and relaxivity studies. The longitudinal relaxivity (r_1_) and transverse relaxivity (r_2_) of the Gd doped *t*-LaVO_4_ (with ***cat***) were determined and compared with that of Gd doped *m*-LaVO_4_ nanoparticles (without ***cat***) (Fig. 10). It is clear from the Fig. 10 that tetragonal phase had better positive contrast enhancement than that of monoclinic phase. Gd doped *t*-LaVO_4_ nanoparticles had an r_1_ of 0.142 mM^−1^s^−1^, which was more than five times that of Gd doped *m*-LaVO_4_ nanoparticles (0.030 mM^−1^s^−1^). The ratio between transverse and longitudinal relaxivity (r_2_/r_1_) was found to be low for Gd doped *t*-LaVO_4_ nanoparticles (2.55), compared to that of Gd doped *m*-LaVO_4_ nanoparticles (5.2). This increase in the r_1_ value coupled with a reduction in r_2_/r_1_ provides for the Gd doped *t*-LaVO_4_ nanoparticles being ideal for use as T_1_ contrast agent^39^,^40^.

**Figure 11:**
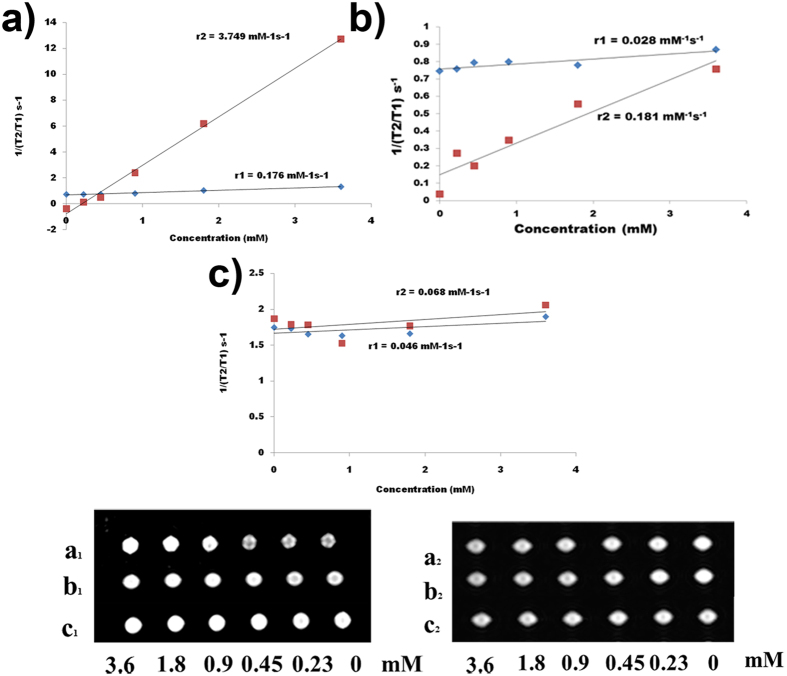
Linear fit plot employed for the calculation of r_1_ and r_2_: T_1_- and T_2_-weighted phantom images of GL nanoparticles with different concentrations where (**a**) 01GL, (**b**) 5GL and (**c**) 1GL. Subscript 1 and 2 represents T_1_ and T_2_ relaxivity.

In order to understand the role of anisotropic morphology, lanthanum chloride was employed as a precursor at 180 °C for 24 h. The results presented in Fig. 11 and Table 3 indicates a variation in the r_2_/r_1_ values. Interestingly at low ***cat*** concentration (0.01 mM), nanoparticles exhibited properties ideal for a T_2_ contrast agents with high r_2_ value (3.749 mM^−1^s^−1^) and r_2_/r_1_ of 21.30^39^,^41^. At 0.05 mM of catechin, nanoparticles lose their ability as T_2_ instead to T_1_ contrast agent with a decrease in the r_2_/r_1_ ratio (6.46) and r_1_ and r_2_ values are greatly reduced^42^,^43^. At a higher cat concentration, SP nanoparticles with almost identical r_1_ (0.046 mM^−1^s^−1^) and r_2_ (0.068 mM^−1^s^−1^) values and moderate r_2_/r_1_ (1.48) ratio, with potential to serve as an excellent candidate for T_1_-T_2_ dual-mode contrast were obtained. This observation is further supported by phantom imaging studies (Fig. 11)^39^,^44^. To conclude, based on the r_2_/r_1_ ratio, r_1_, and r_2_ values, it has been found that the Gd-doped LaVO_4_ nanoparticles developed in this study can be tailored to function as T_1_, T_2_ and T_1_-T_2_ contrast agents through tuning of cat concentration. Such multi-contrast MRI labeling provides unique opportunities for non-invasive multicellular tracking.

## Conclusion

In this paper, we have synthesized Gd-doped LaVO_4_ nanoparticles with different crystal structure and varying morphology, viz., sphere, rods, and irregular rectangular nanocrystals by a catechin directed hydrothermal method. With catechin concentration, the saturation magnetization values of rod shaped Gd-doped LaVO_4_ was greater than that with spherical shape. During this process, the magnetic properties shifted to superparamagnetism from paramagnetism, owing to catechin strong coordination. The direct result of catechin concentration to magnetic property had a remarkable role in MRI applications. MRI studies established that superparamagnetic Gd-doped LaVO_4_ could be employed as both T_1_ and T_2_ contrast agent, as against the common perspective of the same as a T_1_ contrast agent alone.

## Methods

### Synthesis of Gd contrasts with different crystal structure

Gd doped LaVO_4_ (GL) nanoparticles were prepared by co-precipitation method followed by ligand assisted hydrothermal method, carried out according to previously published methods^7^. For tetragonal LaVO_4_ synthesis, we used an efficient ligand- catechin hydrate as a phase transfer agent. 0.06 mmol of catechin hydrate (molar ratio of catechin is 0.05 with respective to La^3+^ ions) was dissolved in 10 mL of double distilled water, to which molar ratio (1:0.05) of La(NO_3_)_3_. 6H_2_O and Gd (NO_3_)_3_. 6H_2_O aqueous solutions were added in drops and kept stirring for 30 min. To that, 1.2 mmol of the Na_3_VO_4_ solution was added in drops resulted in the brown color precipitate. The pH of the brown color precipitate was adjusted to 7. The reaction mixture was autoclaved at 210 °C for 4 h, and the resultant product was washed thrice with water and ethanol twice by centrifugation (1500 rpm for 15 min). It was then air-dried to get the desired product. For monoclinic phase, the same procedure was adopted without catechin hydrate^7^.

### Synthesis of Gd contrasts with varying concentration of catechin hydrate

Different concentrations of catechin hydrate (0.01, 0.05, 0.1 mmol) in 10 mL of distilled water, 1 mmol of LaCl_3_.7H_2_O and Gd(NO_3_)_3_. 6H_2_O (molar ratio = 1:0.05) was added in drops and left stirring for 30 min. Then, 1.05 mmol of Na_3_VO_4_ solution added in drops and stirred for 10 mins to get a brown color precipitate. The resulting solution undergoes hydrothermal treatment at 180 °C for 24 h followed by centrifugation (1500 rpm for 15 min) with double distilled water thrice and twice with ethanol. The final product was obtained by air drying.

The slow-scan powder XRD data for five Gd doped LaVO_4_ nanoparticles, were collected with a step size of 0.01° in the 2θ range of 10–80°. The GSAS-EXPGUI58 program was used for the Rietveld structure refinement from the powder XRD data. The refined parameters were scale factor, background as Chebyshev polynomial, unit cell parameters, profile function (Gaussian and Lorentzian parameters, sample displacement) and atomic positions. The initial structural models for five Gd doped LaVO_4_, were based on their single crystal X-ray structures. The single crystal X-ray structure of Monoclinic LaVO_4_ was used as a structure model for MGL. The single crystal X-ray structure of Tetragonal LaVO_4_ was used as a structure model for TGL, 01GL, 5GL, and 1GL. The structural models turned out to be the correct ones in all cases. For all atoms, the isotropic thermal parameters from the single crystal X-ray structure were used and not refined. Positional parameters and profile functions were refined in alternate cycles until no substantial changes were observed in the positional parameters. The structure refinement proceeded smoothly to yield acceptable agreement factors.

Lattice strain was calculated by Williamson-Hall (W-H) method^45^. A positive slope denotes tensile strain, and a negative slope of the W-H plot denotes compressive strain. A very low lattice strain observed owing to the effective ionic radii mismatch between La^3+^ and Gd^3+^ ions.

### Measurement of magnetic resonance relaxivities

MR relaxivities of GL nanoparticles were measured using a clinical 1.5 T MR scanner (MAGNETOM Avento Tim System, M/s. Siemens, Germany) equipped with a head coil. For this, phantoms of different concentration of GL (0–3.6 mM) were prepared in deionized water and used. For T_2_ relaxometry calculations, a modified T_2_ relaxometry spin echo sequence with TE varying from 15–120 ms with Repetition Time (TR) of 2000 ms were run at three different planes of the phantoms and the pixel intensity with respect to concentration extracted. From the pixel intensity output, the transverse relaxation for each concentration was calculated by employing a linear fit program. For T_1_ measurements, an inversion-recovery sequence was used with 7 non-equidistant time delays of 50, 100, 300, 700, 1200, 2000 and 3000 ms between inversion and the first 90° excitation pulse. Time of Echo (TE) and Time of Repetition (TR) are chosen as 15 and 4000 ms respectively. From the MR images corresponding to these inversion times, signal intensities for all the T_1_ were obtained. The T_1_ relaxation time of each sample was calculated applying these data to the intensity function of the MR signal.

## Additional Information

**How to cite this article**: Vairapperumal, T. *et al*. Catechin tuned magnetism of Gd-doped orthovanadate through morphology as T_1_-T_2_ MRI contrast agents. *Sci. Rep.* **6**, 34976; doi: 10.1038/srep34976 (2016).

## Supplementary Material

Supplementary Information

## Figures and Tables

**Table 1:** Summary of the experimental conditions, crystal lattice parameters and the corresponding size of the GL nanoparticles.

Sample	Catechin molar ratio	Temperature (°C)	Duration (h)	Lattice parameters (Å)	Cell volume (Å^3^)	Crystallite size (nm)	Number mean diameter d (nm)	PDI
MGL	0	210	4	a = 7.0168 Å; b = 7.2518 Å; c = 6.6957 Å	329.31	8.82	798	0.59
TGL	0.05	210	4	a = b = 7.4306 Å; c = 6.5236 Å	360.19	13.46	31.03	0.24
01GL	0.01	180	24	a = b = 7.4066 Å; c = 6.5088 Å	357.06	6.84	68.99	0.33
5GL	0.05	180	24	a = b = 7.4464 Å; c = 6.5247 Å	361.79	16.4	65.92	0.27
1GL	0.1	180	24	a = b = 7.4465 Å; c = 6.5321 Å	362.21	13.32	32.57	0.27

**Table 2:** Magnetic characteristics of the GL nanoparticles.

Sample	*M*_*s*_ (emu/g)	*M*_r_*10^−3^ (emu/g)	H_c_ (G)	S = *M*_*r*_ /*M*_*s*_
MGL	42.4	2.03	525.71	5*10^−5^
TGL	18.32	0.36	137.30	2*10^−5^
01GL	56.19	2.55	441.46	5*10^−5^
5GL	58.87	2.67	441.82	5*10^−5^
1GL	0.0288	3.94	474.08	13.68*10^−2^

**Table 3:** Summary of r_1_, r_2_, and r_2_/r_1_ ratios of the GL nanoparticles.

Sample	magnetism	r_2_(mM^−1^s^−1^)	r_1_(mM^−1^s^−1^)	r_2_/r_1_
MGL	P	0.156	0.03	5.2
TGL	P	0.362	0.142	2.55
01GL	P	3.749	0.176	21.30
5GL	P	0.181	0.028	6.46
1GL	SP	0.068	0.046	1.48
