# Supplementary material for: Catechin tuned magnetism of Gd-doped orthovanadate through morphology as T1-T2 MRI contrast agents
Source: Sci Rep. 2016 Oct 18;6:34976. doi: 10.1038/srep34976 (PMC5067596; doi:10.1038/srep34976)

Supporting Information

Catechin tuned magnetism of Gd-doped orthovanadate through morphology as T_1-T_2 MRI contrast agents

Vairapperumal Tamilmani,[a] Ariya Saraswathy[b], Ramapurath S. Jayasree[c], Kalarical Janardhanan Sreeram[a]*, and Balachandran Unni Nair [a]

a. Chemical laboratory, CSIR-Central Leather Research Institute, Adyar, Chennai, 600 020, India.

b. Department of Physics, NSS College, Pandalam 689501, Kerala, India

c. Biophotonics and Imaging Lab, Sree Chitra Tirunal Institute for Medical Sciences and Technology, BMT Wing, Poojappura, Trivandrum 695012, India.

Figure S1. Cell viability (A) and representative image of cells (B) estimated by MTT assay in HaCaT cell lines cultured in the presence of 0−100 µG/mL TGL at 37 °C for 24 h. Scale bar represents 100µM

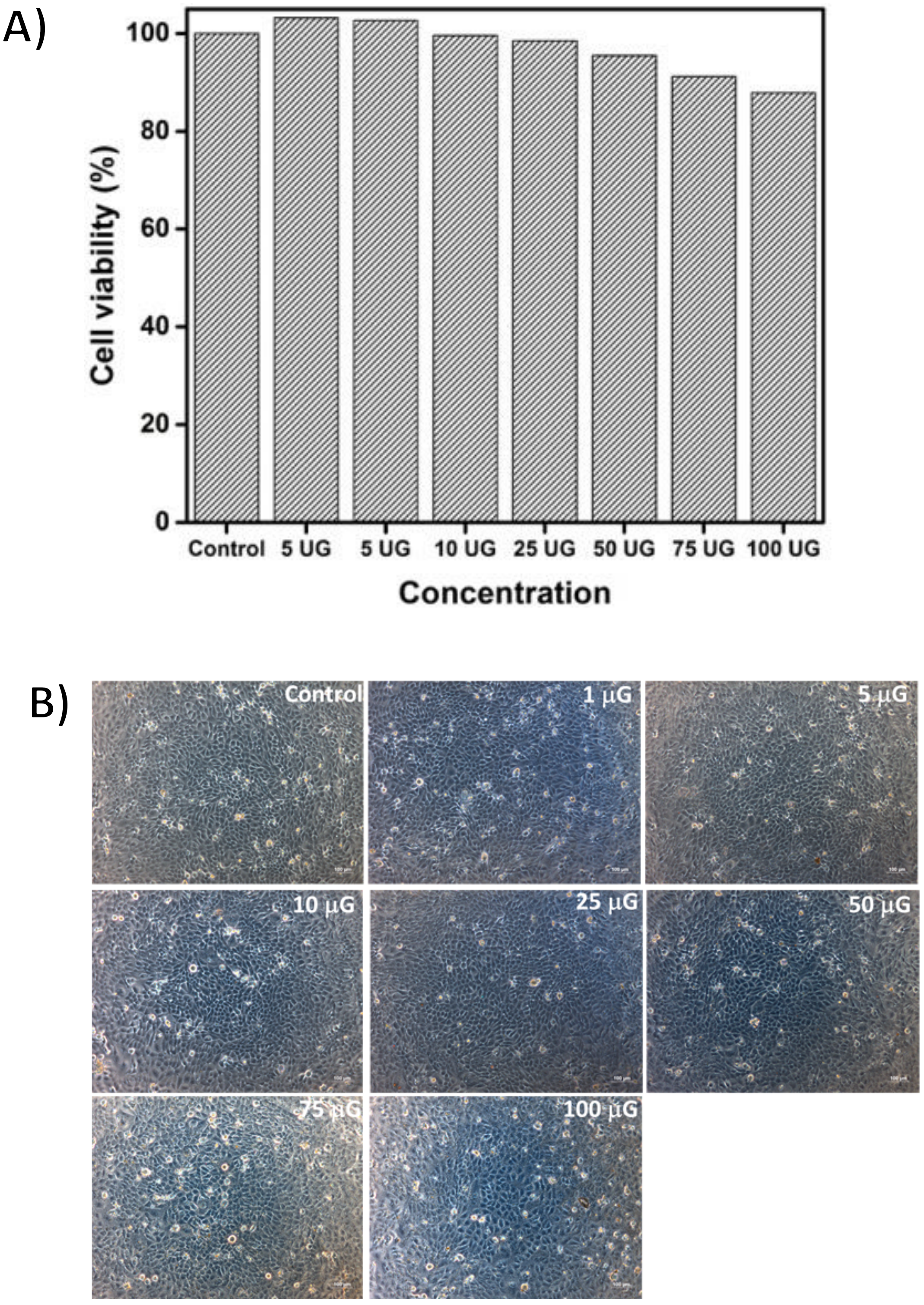

Supplement: Supplementary Information [file srep34976-s1.pdf]
